# Draft Genome Sequence of Enterobacter roggenkampii Strain OS53, Isolated from Corroded Pipework at an Offshore Oil Production Facility

**DOI:** 10.1128/MRA.00583-20

**Published:** 2020-08-20

**Authors:** Silvia J. Salgar-Chaparro, Genis Castillo-Villamizar, Anja Poehlein, Rolf Daniel, Laura L. Machuca

**Affiliations:** a Curtin Corrosion Centre, WA School of Mines: Minerals, Energy and Chemical Engineering, Curtin University, Bentley, WA, Australia; b Genomic and Applied Microbiology & Göttingen Genomics Laboratory, Institute of Microbiology and Genetics, Georg-August University Göttingen, Göttingen, Germany; c Línea Tecnológica Biocorrosión, Corporación para la Investigación de la Corrosión C.I.C. Piedecuesta, Santander, Colombia; University of Delaware

## Abstract

Here, we report the genome sequence of Enterobacter roggenkampii strain OS53, isolated from corroded pipework at an offshore oil production facility. The draft genome sequence comprises 6 contigs and contains 5,194,507 bp with an average GC content of 55.90%.

## ANNOUNCEMENT

Enterobacter roggenkampii is a facultative, anaerobic, rod-shaped, Gram-negative bacterium that belongs to the family *Enterobacteriaceae*. Most *Enterobacter* spp. can produce organic acids from their metabolic activities (1). Acid-producing microorganisms promote microbiologically influenced corrosion by local acidification (2, 3). *E. roggenkampii* strain OS53 was isolated from corrosion products formed on corroded pipework at an Australian offshore oil production facility. Tubes containing sterile culture medium for sulfide-producing prokaryotes (SPP) (4) were inoculated with corrosion products and incubated at 40°C under anaerobic conditions. OS53 was isolated using a streaking technique with plates prepared with the same SPP medium composition and 15 g/liter agar-agar. Plates were incubated in anaerobic jars with AnaeroGen sachets (Oxoid). Individual colonies were restreaked onto SPP agar until they were axenic, as determined by microscopy.

Single colonies were transferred to SPP broth and grown overnight at 40°C for DNA extraction with a DNeasy PowerSoil kit (Qiagen). Extracted DNA was used for both Illumina and Nanopore sequencing. The Illumina library was prepared with the Nextera XT DNA sample preparation kit, and paired-end sequencing was performed using the MiSeq reagent kit v3 (600 cycles) and the MiSeq instrument as described by the manufacturer (Illumina, San Diego, CA, USA). The library for Nanopore sequencing was prepared using the one-dimensional (1D) genomic DNA sequencing protocol (SQK-LSK109) without any size selection. The library was loaded on a SpotON flow cell Mk I (R9.4) and sequenced with a MinION device (Oxford Nanopore), and reads were base called using Albacore v2.3.1. After quality filtering using fastp v0.19.4 (5), totals of 1,077,509 long reads (Nanopore) with an average length of 2,769 bp and 2,630,600 short reads (Illumina) with an average length of 281 bp were used for the assembly. Sequences were assembled *de novo* using a hybrid assembly strategy with Unicycler v0.4.7 (6). The assembly comprised 6 contigs in total. The largest contig length was 4,916,357 bp and covered 94.6% of the total assembled genome sequences. The draft genome sequence is 5,194,507 bp long with an average GC content of 55.90% and coverage of 147-fold. The assembly was visualized and validated using Bandage v0.8.1 (7). Default parameters were used for all software tools unless otherwise noted.

Genome annotation with the NCBI Prokaryotic Genome Annotation Pipeline v4.10 (8) predicted 5,067 genes, including 4,859 protein-coding genes with predicted functions, 91 genes coding for hypothetical proteins, and 86 tRNA, 25 rRNA, and 6 noncoding RNA (ncRNA) genes. Average nucleotide identity (ANI) was calculated using pyANI v0.2.7 (9). It was found that OS53 is closely related to *E. roggenkampii* strain DSM 16690 (GenBank accession no. CP017184) with an ANI value of 98.62%.

The metabolic pathway identification was carried out using the KEGG Automated Annotation Server (KAAS) (10). The analysis revealed that the genome possesses an entire set of genes for glycolysis, tricarboxylic acid cycle, pentose phosphate, and fatty acid biosynthesis and degradation. Other genes potentially involved in corrosion reactions were also detected.

### Data availability.

This genome sequence was submitted to GenBank under accession no. JAACJF000000000. The raw reads have been deposited in the NCBI SRA database under accession no. SRR11492388 and SRR11492389.
